# Supplementary material for: Exploring Caloric Restriction in Inpatients with Eating Disorders: Cross-Sectional and Longitudinal Associations with Body Dissatisfaction, Body Avoidance, Clinical Factors, and Psychopathology
Source: Nutrients. 2023 Jul 31;15(15):3409. doi: 10.3390/nu15153409 (PMC10420884; doi:10.3390/nu15153409)

Exploring Caloric Restriction in Inpatients with Eating Disorders: Cross-Sectional and Longitudinal Associations with Body Dissatisfaction, Body Avoidance, Clinical Factors, and Psychopathology

Supplementary Material

Supplementary Figure S1. Model component selection for BSQ

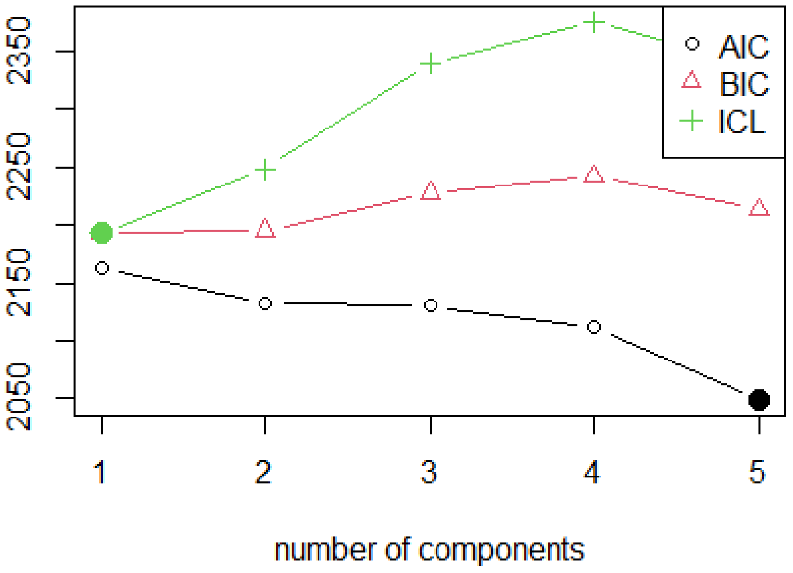

Supplementary Figure S2. Correlations in the whole sample with Holm correction for p-values

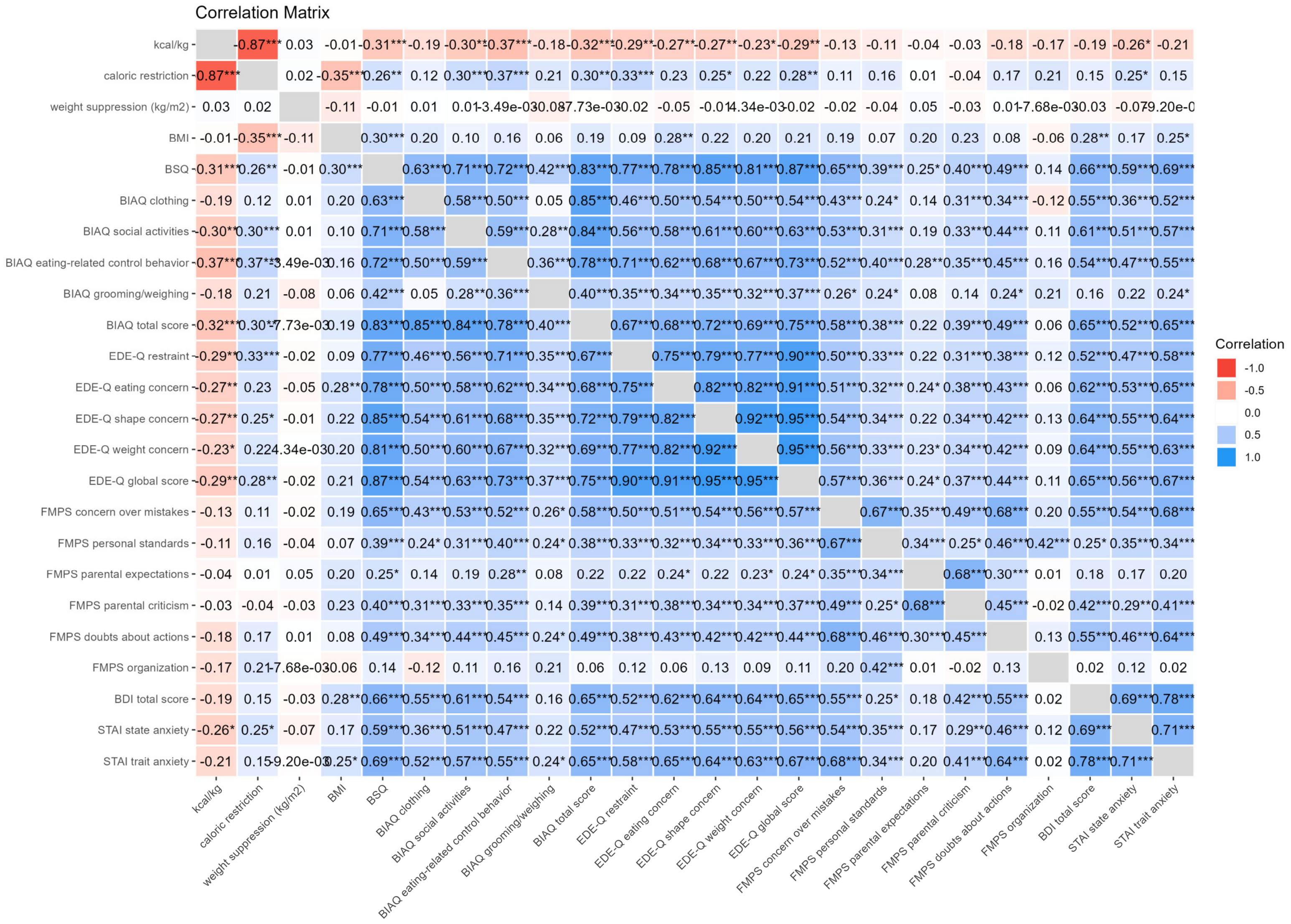

Supplementary Figure S3. Model check for BSQ ~caloric restriction*weight suppression (kg/m2)+BMI+binge-purging symptoms+age (yrs)+duration of illness (yrs)

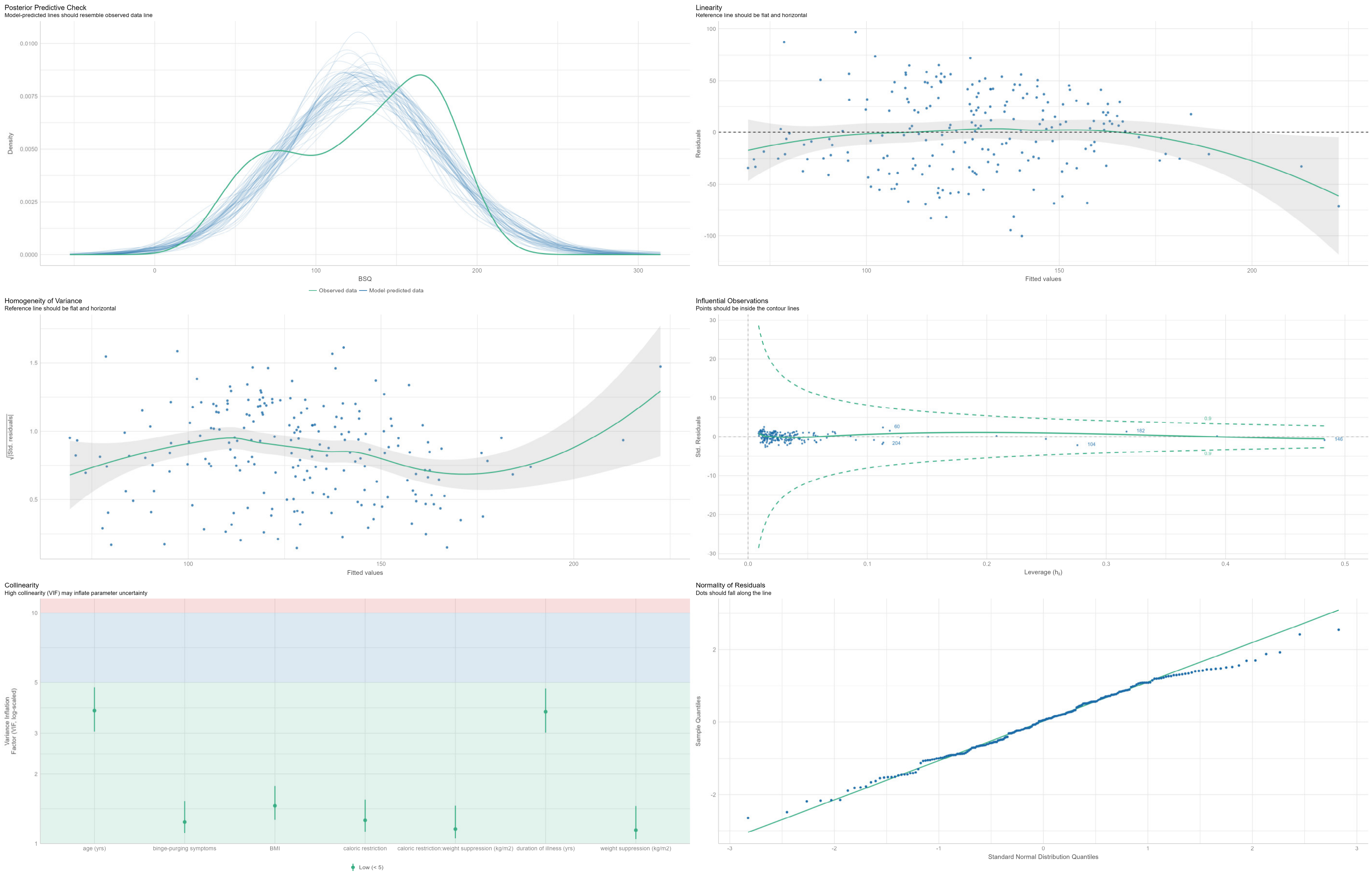

Supplementary Figure S4. Model check for BIAQ total ~caloric restriction*weight suppression (kg/m2)+BMI+binge-purging symptoms+age (yrs)+duration of illness (yrs)

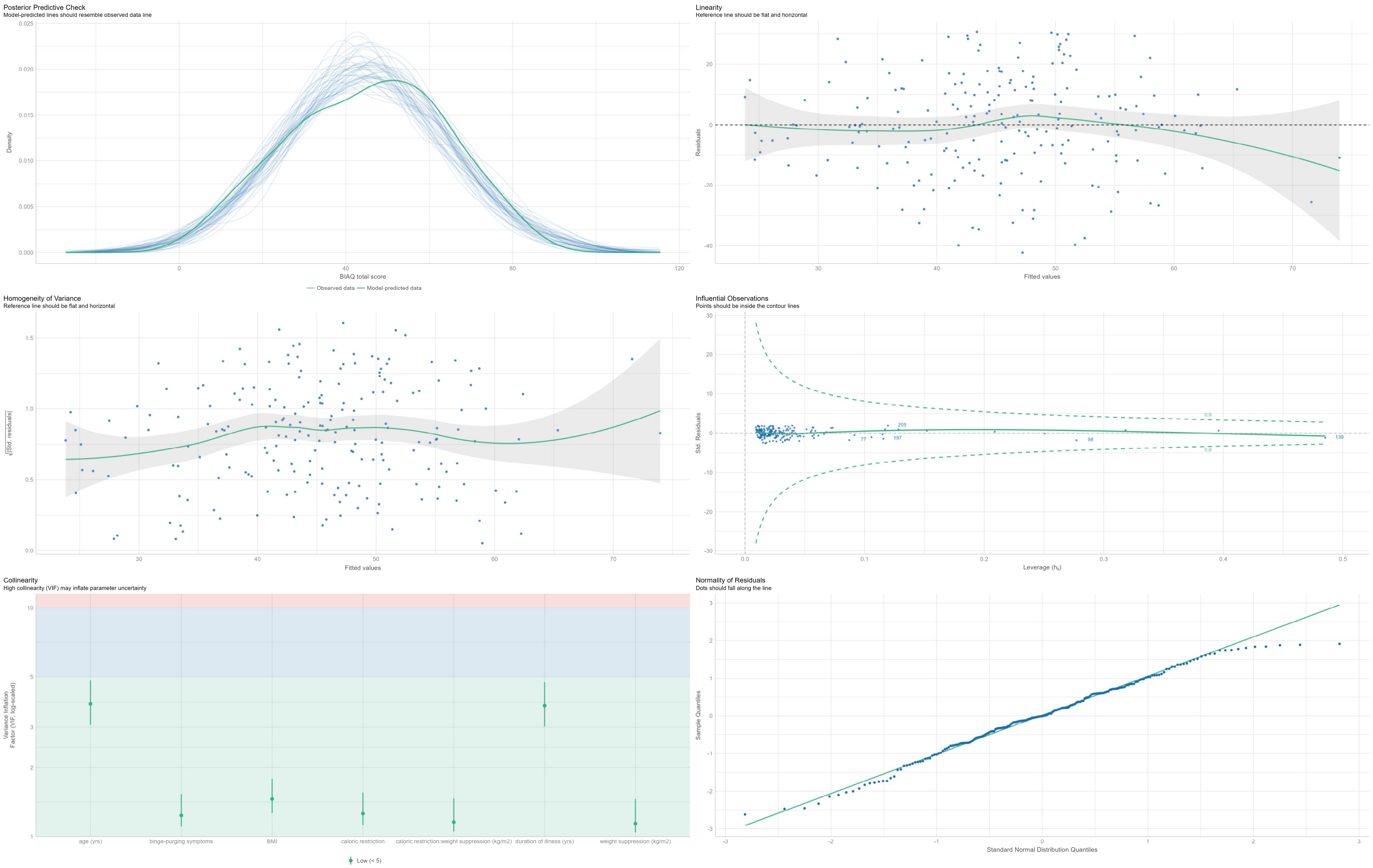

Supplementary Table S1. Variable importance from random forests for caloric intake at discharge

	Variable importance (root MSE of predicted versus permuted)
BMR	2.544
length of stay (days)	2.107
BMI	1.907
daily calorie need	1.639
FMPS concern over mistakes	1.523
kcal/kg	1.387
caloric restriction	1.106
BMI EOT	1.072
STAI state anxiety	0.957
EDE-Q eating concern	0.847
EDE-Q global score	0.844
BDI total score	0.755
BSQ	0.736
EDE-Q weight concern	0.691
BIAQ eating-related control behavior	0.606
EDE-Q shape concern	0.585
enteral therapy	0.576
weight suppression	0.464
binge-purging symptoms	0.457
BIAQ social activities	0.438
STAI trait anxiety	0.353
BIAQ total score	0.344
duration of illness (yrs)	0.333
FMPS parental criticism	0.266
EDE-Q restraint	0.225
FMPS parental expectations	0.200
history of overweight/obesity	0.136

	Variable importance (root MSE of predicted versus permuted)
BIAQ grooming/weighing	0.117
sex	0.000
FMPS personal standards	0.000
age (yrs)	0.000
FMPS organization	0.000
FMPS doubts about actions	0.000
activity	0.000
BIAQ clothing	0.000
diagnosis	0.000

Supplementary Table S2. Variable importance from random forests for caloric restriction at discharge

	Variable importance (root MSE of predicted versus permuted)
daily calorie need	6.539
FMPS concern over mistakes	5.036
STAI state anxiety	4.651
BMR	4.280
EDE-Q global score	4.092
BSQ	3.844
age (yrs)	3.634
activity	3.245
length of stay (days)	3.200
EDE-Q weight concern	3.061
EDE-Q eating concern	2.773
EDE-Q shape concern	2.725
FMPS personal standards	2.449
caloric restriction	2.186
duration of illness (yrs)	2.072
STAI trait anxiety	1.903
EDE-Q restraint	1.897
BMI EOT	1.812
BDI total score	1.600
BIAQ eating-related control behavior	1.578
BIAQ grooming/weighing	1.437
enteral therapy	1.044
BIAQ total score	0.881
BIAQ social activities	0.715
FMPS parental expectations	0.504
binge-purging symptoms	0.384
FMPS doubts about actions	0.373

	Variable importance (root MSE of predicted versus permuted)
FMPS organization	0.317
sex	0.200
kcal/kg	0.000
history of overweight/obesity	0.000
weight suppression	0.000
BMI	0.000
diagnosis	0.000
BIAQ clothing	0.000
FMPS parental criticism	0.000

Supplementary Figure S5. Model check for kcal/kg EOT ~ BMR + length of stay (days) + BMI + daily calorie need + FMPS concern over mistakes

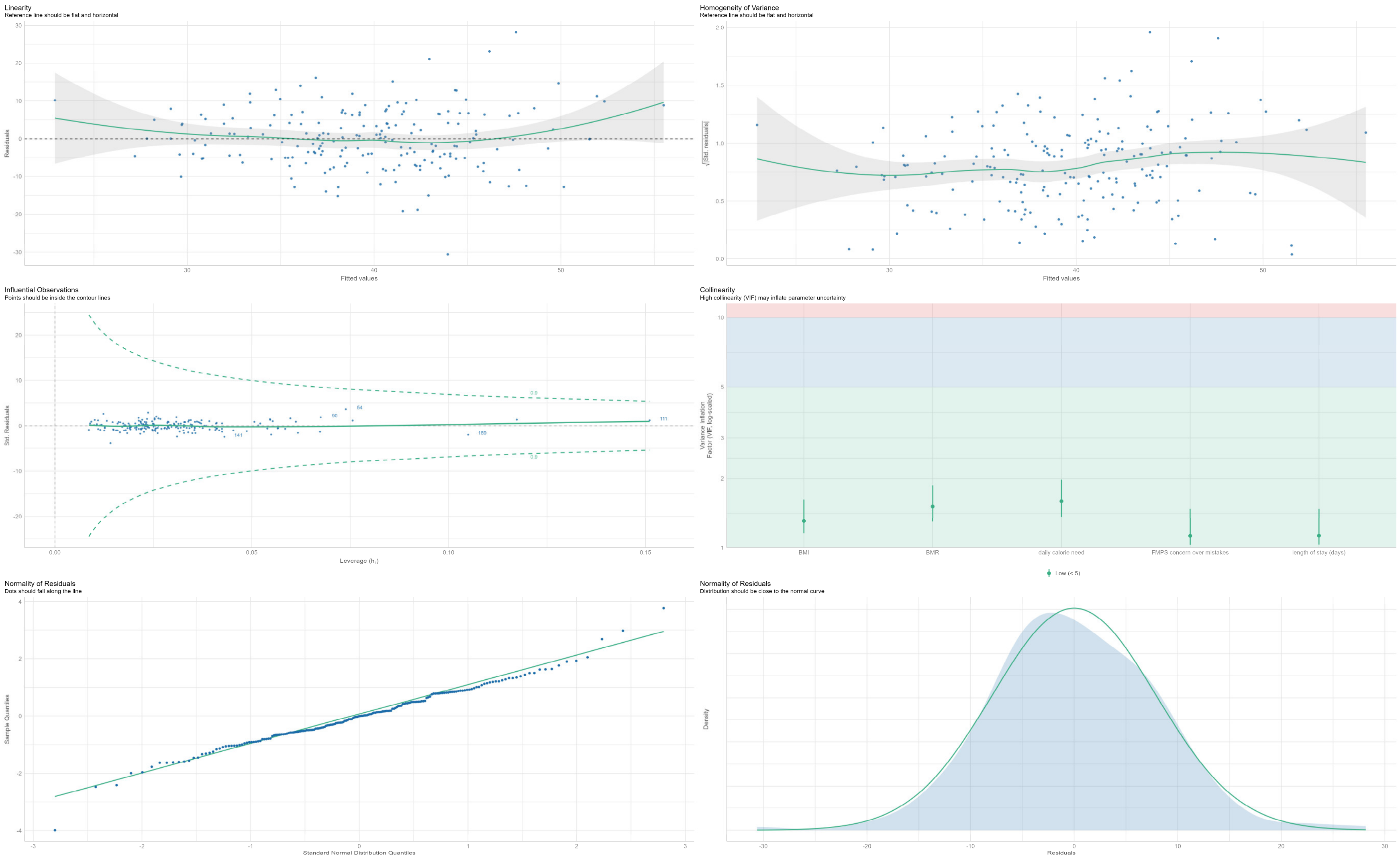

Supplementary Figure S6. Model check for caloric restriction EOT ~ daily calorie need + FMPS concern over mistakes + STAI state anxiety + BMR + EDE-Q global score

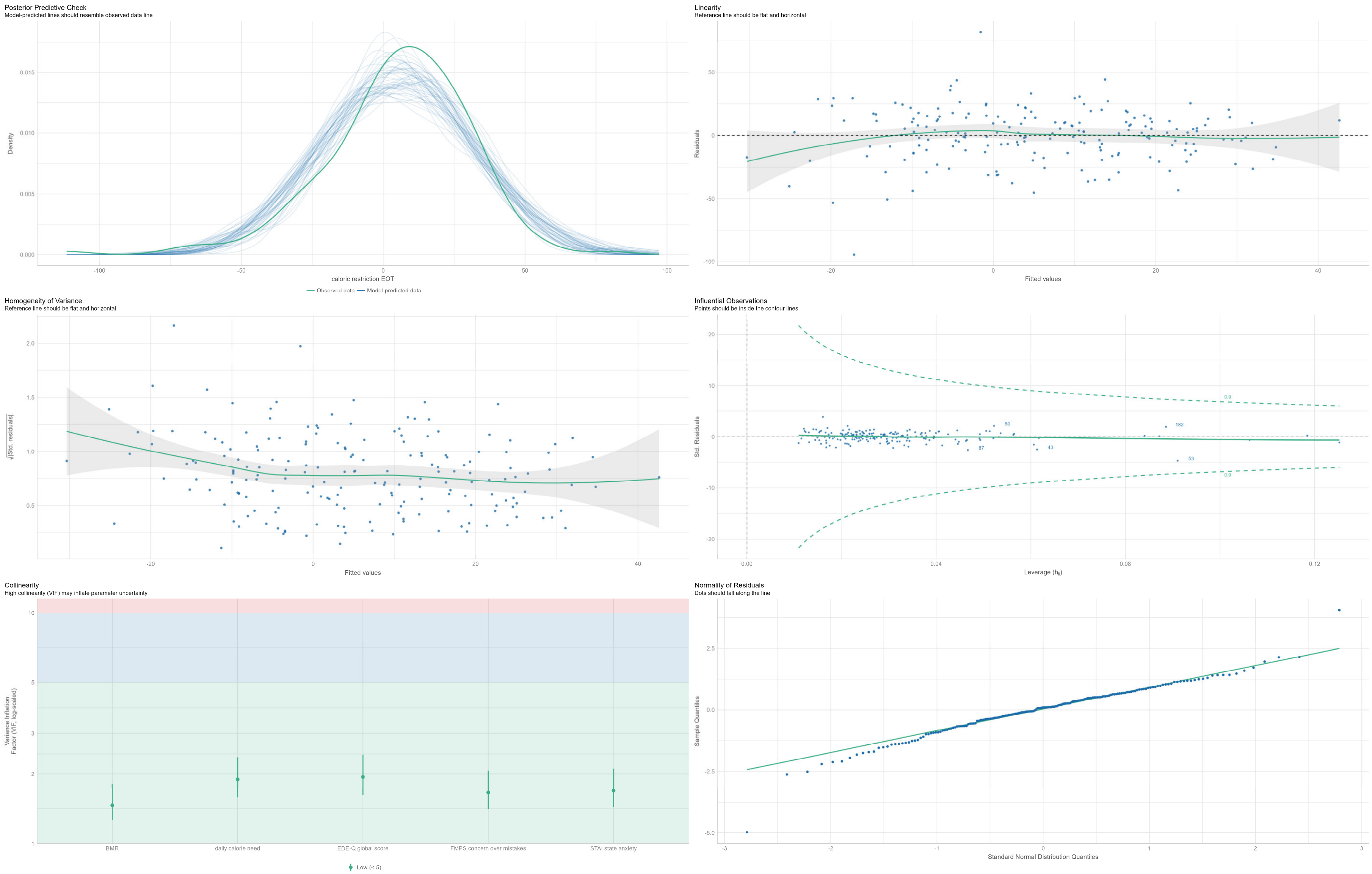

Supplement: Supplementary file 1 [file nutrients-15-03409-s001.zip › nutrients-2509313-supplementary.pdf]
